# Engineered TtgR-Based Whole-Cell Biosensors for Quantitative and Selective Monitoring of Bioactive Compounds

**DOI:** 10.3390/bios15080554

**Published:** 2025-08-21

**Authors:** Kyeongseok Song, Haekang Ji, Jiwon Lee, Geupil Jang, Youngdae Yoon

**Affiliations:** 1 Department of Environmental Health Science, Konkuk University, Seoul 05029, Republic of Korea; kssong1999@konkuk.ac.kr (K.S.); dokijiwon@konkuk.ac.kr (J.L.); 2 School of Biological Sciences and Technology, Chonnam National University, Gwangju 61186, Republic of Korea; yk3@chonnam.ac.kr

**Keywords:** transcription factor-based biosensor, TtgR, flavonoid detection, structure-guided protein engineering, whole-cell sensor

## Abstract

TtgR, a transcriptional repressor from *Pseudomonas putida*, plays a key role in regulating multidrug resistance by controlling the expression of genes in response to various ligands. Despite its broad specificity, TtgR represents a promising candidate for the development of transcription factor (TF)-based biosensors. In this study, we utilized TtgR and its native promoter region (P*_ttgABC_*) as genetic components to construct TF-based biosensors in *Escherichia coli*. By coupling TtgR and P*_ttgABC_* with *egfp*, we developed a biosensor responsive to diverse flavonoids. To enhance the selectivity and specificity of the biosensor, we genetically engineered a TtgR-binding pocket. Engineered TtgR variants exhibited altered sensing profiles, enabling the development of biosensors with tailored ligand responses. Computational structural analysis and ligand docking provided insights into the interaction mechanisms between TtgR variants and flavonoids. Notably, biosensors based on wild-type TtgR and its N110F mutant were capable of quantifying resveratrol and quercetin at 0.01 mM with >90% accuracy. Although the precise molecular mechanisms involved remain unclear and further optimization is needed, the biosensors developed herein demonstrate strong potential for applications in numerous fields. This study lays the foundation for future research that could extend the utility of TtgR-based biosensors to synthetic biology, metabolic engineering, and beyond.

## 1. Introduction

Bacteria have innate defense mechanisms that mitigate the effects of toxic compounds in their environment, whereas other organisms, such as plants, produce protective compounds to combat invasive pathogens. Among the tools used in bacterial defense, multidrug resistance (MDR) efflux pumps play a pivotal role by exhibiting broad specificity toward diverse compounds, including antibiotics, biocides, detergents, and organic solvents, thereby contributing to their antimicrobial activity [1,2,3]. These efflux systems are regulated by transcription factors (TFs), such as QacR in *Staphylococcus aureus*, EmrR in *Escherichia coli*, TtgR in *Pseudomonas putida*, and BmrR in *Bacillus subtilis* [4,5,6]. These TFs have evolved broad specificity to enhance bacterial tolerance to diverse toxic compounds. TtgR, a transcriptional regulator, is essential for modulating MDR in *P. putida* DOT-T1E [5]. It represses the expression of the ttgABC efflux pump, which actively expels toxic compounds, such as antibiotics, organic solvents, and flavonoids. Angueld et al. determined the structure of TtgR and revealed its binding dynamics with various ligands, including antibiotics and plant secondary metabolites [7]. Furthermore, the ligand-binding site adapts slightly to different ligands, enabling TtgR to induce expression of the ttgABC efflux pump upon interaction with a target ligand [8,9].

Flavonoids, a diverse group of plant secondary metabolites, are known for their antimicrobial, antioxidant, and anti-inflammatory properties [10,11]. Structurally, they are characterized by a C6–C3–C6 (A–C–B ring) backbone with varying degrees of substitution and saturation [12]. Studies have shown that flavonoids such as naringenin, quercetin, and phloretin exhibit strong interactions with TtgR [9,13,14]. These interactions are mediated by hydrogen bonding and van der Waals forces within the ligand-binding pocket, which features a combination of hydrophobic and hydrophilic residues. The distinct binding dynamics of flavonoids with TtgR further underscore its broad specificity [7,13]. These molecular interactions between flavonoids and TtgR not only highlight the broad ligand recognition capacity of this transcription factor but also provide a rationale for its application in biosensor systems. In particular, the ability of TtgR to respond to structurally diverse flavonoids positions it as a promising molecular recognition element for developing selective, ligand-responsive biosensors. By exploiting this natural specificity and engineering its key binding residues, TtgR-based biosensors can be tailored to achieve improved performance in terms of sensitivity, dynamic range, and selectivity.

Transcription factor (TF)-based biosensors have emerged as powerful tools in synthetic biology and biotechnology due to their ability to convert specific molecular recognition events into gene expression outputs. These biosensors are particularly attractive for use in live-cell systems because of their high specificity, modularity, and adaptability to various input signals. In this regard, TF-based biosensors have been actively investigated over the past few decades, exhibiting applications across diverse scientific fields due to their ability to target specific molecules and regulate gene expression [15,16,17]. Among these TFs, TtgR has attracted attention owing to its promiscuous ligand-binding capability and regulatory responsiveness. However, most natural TtgR-based biosensors reported to date exhibit relatively narrow dynamic ranges and moderate selectivity. Nonetheless, the performance of biosensors based on TtgR could be further improved by engineering multiple components, including the TF itself, promoters, enhancers, and ribosome-binding sites (RBS) [18,19]. For example, a Pb-specific *E. coli* cell-based biosensor was generated by engineering ZntR, a Zn-responsive TF, and its performance was modulated by employing chimeric MerR-family regulators [20,21]. In addition to such specific examples, numerous excellent review articles have highlighted how rational modifications of TF-based biosensor components—including transcription factors, promoters, operator sequences, ribosome-binding sites, and reporter modules—can significantly enhance sensor performance across various applications [19,22,23]. Despite significant advances, the mechanism by which TtgR mutations alter ligand specificity remains poorly understood. Recent computational and experimental studies have provided insights into the structural basis of TtgR–ligand interactions [13,24]. For instance, phloretin forms stable hydrogen bonds with key residues such as Asn110 and Asp172, whereas resveratrol demonstrates high binding affinity, highlighting the promiscuous yet selective nature of TtgR. These findings suggest that engineering TtgR mutants could enable the design of biosensors with enhanced specificity and broader applications.

In this study, we designed and developed *E. coli*-based biosensors using the TtgR regulatory system and enhanced green fluorescent protein (eGFP) as a reporter. This biosensor demonstrated selective responses to flavonoids, with fluorescence intensity variations correlating with the chemical structure of the ligand. Additionally, we explored the functional consequences of TtgR mutations on ligand specificity through computational analysis and assessed the applicability of this system in synthetic biology. Following in silico modeling and structure-guided mutagenesis, the engineered TtgR variants were used to construct biosensors capable of quantitatively detecting quercetin and resveratrol. These biosensors demonstrated their potential as reliable tools for the real-time monitoring of bioactive compounds, including flavonoids and resveratrol. By combining experimental data with computational modeling, we aimed to establish a comprehensive understanding of the role that TtgR plays in ligand recognition and its potential for biotechnological innovation in diverse research fields, such as metabolic engineering and synthetic biology.

## 2. Materials and Methods

### 2.1. Materials

The *E. coli* BL21(DE3) and DH5α strains were used as competent cells for host and gene cloning cells for the biosensors, respectively. Lysogeny broth (LB), consisting of tryptone, yeast extract, and sodium chloride, was used for *E. coli* cultivation and was purchased from Sigma-Aldrich (St. Louis, MO, USA). Restriction enzymes and ligases were purchased from Takara (Otsu, Japan). Hotstar Taq polymerase (Qiagen, Hilden, Germany) was used for gene amplification, and PfuTurbo (Invitrogen, Waltham, MA, USA) used for site-directed mutagenesis. Flavones, flavanones, and resveratrol were purchased from Sigma-Aldrich (Steinheim, Germany) and prepared as 50 mM stock solutions in dimethyl sulfoxide (DMSO). The primers used were synthesized by Macrogen (Seoul, Republic of Korea), and all DNA sequences confirmed through sequencing.

### 2.2. Construction of Plasmids and Escherichia coli Cell-Based Biosensors

The genomic DNA of *P. putida* was extracted using a Genomic DNA Extraction Kit (Bioneer, Daejon, Republic of Korea), following the manufacturer’s protocol, which was then used as a template for amplifying *ttgR* and the corresponding operator sequence via PCR. The amplified genes were purified using a Gel Elution Kit (Bioneer), following the manufacturer’s protocol. The *ttgR* and P*_ttgABC_* fragments were inserted into pCDF-Duet and pZnt-eGFP vectors [25] with NdeI/NotI and BglII/XbaI restriction enzymes to construct the sensing and signal reporting plasmids, named pCDF-TtgR and pTtg-eGFP, respectively. To genetically engineer TtgR, mutations were introduced around its ligand-binding site via site-directed mutagenesis using PfuTurbo and the corresponding primer pairs. Mutagenesis of *ttgR* was verified through DNA sequencing. Then, the pair of plasmids carrying *ttgR*s and P*_ttgABC_:egfp* was introduced into *E. coli* BL21 to construct the *E. coli* cell-based biosensors. Genetic characteristics of the *E. coli* cell-based biosensors are listed in Table 1, and the primer sequences used are listed in Table S1.

### 2.3. Biosensor Assay

**Figure 1:**
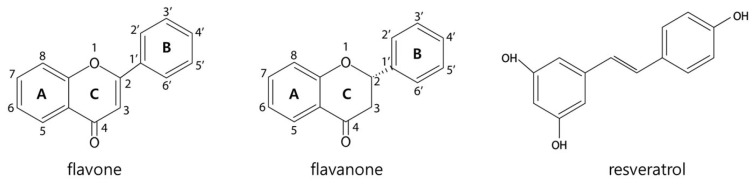
Structural characteristics of the tested flavone, flavanone and resveratrol.

The *E. coli* BL21 strains harboring the sensing and reporter plasmids underwent biosensor assays to elucidate their responses to diverse chemicals, including flavonoids and resveratrol. Briefly, the overnight cultures of biosensor cells were inoculated into fresh Luria–Bertani (LB) media, whereafter the cells were exposed to 0.005–5 mM chemicals when the optical density at 600 nm (OD_600_) reached 0.3 at 37 °C in a shaking incubator (250 rpm). After 1–3 h of exposure, the expression levels of eGFP were measured using a fluorescence spectrometer (FluoroMate FC-2; SCINCO, Seoul, Republic of Korea) with an excitation wavelength of 480 nm. The emission wavelength was measured from 500 to 600 nm, and the values recorded at 510 nm were converted to induction coefficient values, which were defined using the following formula: (eGFP intensity with chemical exposure)/(eGFP intensity without chemical exposure), with compensation for the OD_600_ values. A list of the compounds tested and their structural characteristics are summarized in Figure 1 and Table 2.

### 2.4. In Silico Docking Experiments

For the in silico docking experiments, the crystal structure of TtgR deposited in the PDB database (2uxh.pdb) was selected and modified [7]. Chain A was selected from the dimer structure. The water molecules, original ligand, quercetin, and chain B were removed using PyMOL software (version 2.5.4) to generate the apoprotein for the docking experiments [26]. As a ligand structure, quercetin was also obtained from the PDB database (2uxh.pdb). Polar hydrogens were added to the apo-TtgR protein. Both receptor and ligand structures were saved as pdbqt files using AutoDockTools (version 1.5.7) [27]. A grid box was set to the binding pocket. The center and size of the grid box were (16.0, 29.9, −4.6) and (50, 50, 50), respectively. The ligands were docked to the TtgR wild-type (WT) using AutoDock Vina (version 1.1.2) [28]. To validate the docking method, quercetin was redocked into the apo-TtgR WT. For further structural studies, structures of several ligands and TtgR mutants were generated. Certain residues, including Val96 (valine), Asn110, and His114 (histidine), which are important for ligand docking, were mutated using a mutagenesis module from PyMOL software. After the docking simulation, diverse software programs were used to evaluate the results. Nine docked conformations were generated using AutoDock Vina. All docked conformations were compared with each other, and the protein–ligand complexes obtained using Chimera (version 1.18) [29]. The protein–ligand complexes were visualized and analyzed using PyMOL and LigPlot (version 2.2.9) [30].

### 2.5. Application of Escherichia coli Cell-Based Biosensors in Quercetin and Resveratrol Quantification

To elucidate the applicability of the *ttg* operon system-based biosensors, they were applied to quantify quercetin and resveratrol. The biosensors with TtgRs showing superior specificity for quercetin and resveratrol (biosensor-TtgR N110F and biosensor-TtgR WT) were selected for quantification. The biosensors were exposed to 0–5 mM chemicals to construct standard curves, whereafter the concentrations of unknown samples were calculated from the standard curves. The same samples were analyzed using a YL9100 Plus high-performance liquid chromatography (HPLC) system (Youngin Chromass Co., Ltd., Anyang, Republic of Korea) equipped with a ZORBAX Eclipse XDB-C18 reversed-phase column (4.6 × 150 mm; Agilent Technologies, Santa Clara, CA, USA) and photodiode array detector. For the analytical runs, the mobile phase consisted of water with 0.1% formic acid and acetonitrile, with the following gradient program: 20% acetonitrile at 0 min, 70% at 10 min, and 100% at 15 min. The flow rate was set to 1 mL/min, and UV detection was performed at 245 nm. Results of the biosensor assay and HPLC analysis were compared to validate detection accuracy.

### 2.6. Statistical Analysis

All experimental data were obtained from more than triplicated experiments, and the values are indicated as the mean and standard deviation. Statistical analysis and data validation were performed using the R version 4.3.0 package DescTools version 0.99.59 [31,32]. Significant differences in the data compared to the control were validated using Dunnett’s test.

## 3. Results

### 3.1. Sensing Mechanism of Escherichia coli Cell-Based Biosensors Employing the ttg Operon System

**Figure 2:**
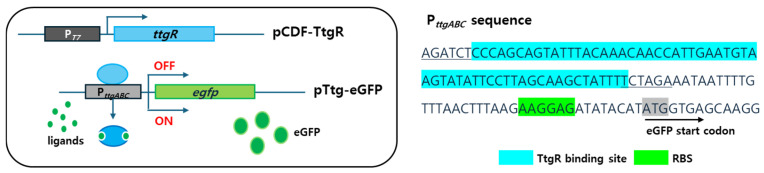
Schematic diagram of the *Escherichia coli* cell-based biosensors based on the *ttg* operon system and promoter sequences of *ttgABC*. The promoter region includes the ribosome-binding site and start codon of *egfp*.

To construct biosensors based on the *ttg* operon system, the TF, TtgR, and promoter region of *ttgABC* fused with *egfp* were employed as target sensing and signal reporting elements, respectively. The amplified *ttgR* gene and P*_ttgABC_:egfp* were inserted into pCDF-Duet and pET-21a(+) vectors, respectively, and introduced into *E. coli* BL21. As shown in Figure 2, the expression of eGFP was repressed by TtgR in the absence of ligands; however, it was induced in the presence of ligands because TtgR was released upon interacting with them. Therefore, the ligand concentrations were quantified by measuring the expression levels of eGFP. The sequences, including P*_ttgABC_*, are shown in Figure 2, along with the RBS and start codon of *egfp*. The *E. coli* cell-based biosensor based on the *ttg* operon system showed constitutive expression of eGFP without TtgR but repressed expression with TtgR. These results indicated that TtgR acts as a repressor in the *ttg* operon and regulates the expression of genes located downstream of the promoter region. Therefore, the genetic engineering of TtgR could modulate the characteristics of biosensors employing the *ttg* operon system.

### 3.2. Characteristics of Escherichia coli Cell-Based Biosensors Employing the TtgR Wild-Type

**Figure 3:**
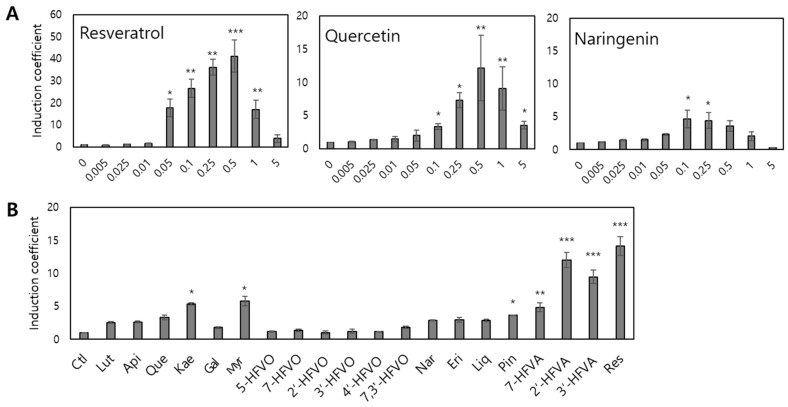
Responses of the biosensor-TtgR wild-type to the tested compounds. (**A**) Dose-dependent fluorescence responses to resveratrol, quercetin, and naringenin (0–5 mM). Compound concentration (mM) is shown on the x-axis. (**B**) Relative fluorescence responses (induction coefficient values) at 0.1 mM of each compound. The data were replicated more than three times, and asterisks indicate significant differences in data compared to the control (* *p* ≤ 0.05, ** *p* ≤ 0.01, and *** *p* ≤ 0.001 using Dunnett’s test).

TtgR acts as a regulatory protein for the expression of *egfp* located downstream of P*_ttgABC_*. As TtgR is a TF with broad ligand specificity for diverse flavonoids, antibiotics, and resveratrol, elucidating the target selectivity and specificity of *E. coli* cell-based biosensors employing the *ttg* operon system is pivotal. Resveratrol, quercetin, and naringenin interact with TtgR; therefore, these compounds were subjected to the biosensor assay [7,33]. The *E. coli* BL21 strain harboring the *ttgR* WT and P*_ttgABC_*:*egfp*, named biosensor-TtgR WT, was inoculated with 5 mL fresh LB and exposed to 0–5 mM resveratrol, quercetin, and naringenin at an OD_600_ of 0.3. After 2 h of exposure, the expression levels of eGFP were measured using a fluorescence spectrometer, and the arbitrary units of fluorescence were converted to induction coefficient values. As shown in Figure 3A, the biosensor exhibited increased fluorescence signals in a concentration-dependent manner, with resveratrol showing the strongest response among all the tested chemicals. In addition, flavonoids at concentrations > 0.5 mM showed decreased induction coefficient values because the growth of *E. coli* was inhibited by flavonoids. Although 0.25 and 0.5 mM chemical exposure showed stronger signals, 0.1 mM was selected for further investigation to exclude chemical toxicity in the *E. coli* cells.

To elucidate the specificity and selectivity, the biosensor-TtgR WT was exposed to 0.1 mM of the chemicals listed in Table 2, and the results are shown in Figure 3B. As expected, the biosensor-TtgR WT showed the strongest response to resveratrol, and flavones (including quercetin and kaempferol) showed stronger signals than others. Among the flavanones, those with a single hydroxyl group on the B ring, 2′- and 3′-hydroxyflavanone (HFVA) showed strong responses comparable with that of resveratrol. Although flavones and flavanones share structural similarities, the double bond between C2-C3 in the B ring of flavones would cause differences in signals. Thus, the rigidity of flavonoids might play a critical role in their interaction with TtgR.

### 3.3. Genetic Engineering of TtgR

The target selectivity and specificity of TF-based biosensors are determined by the combined effects of several genetic factors, including the nature of TFs, strength of promoters and operators, number of operators, and RBS [18,34,35]. To modulate the characteristics of biosensors based on the *ttg* operon system, we targeted TtgR, a TF that regulates the *ttg* operon, for genetic engineering based on an analysis of the three-dimensional structure of TtgR deposited in PDB [7]. In the crystal structure of TtgR, the A and C rings of quercetin interacted with the hydrophobic residues of TtgR (Val96, Ile141, and Phe168), whereas the B ring of quercetin interacted with the polar residues of TtgR (Asn110 and His114). TtgR forms a dimer that plays the role of a TF with two ligands located at both binding sites of the monomers (Figure S1). To modulate the biosensor characteristics, the amino acids involved in ligand binding were targeted for mutagenesis. To alter properties including hydrophobicity and size, Val96, Asn110, His114, Ile141, and Phe168 were mutated to different amino acids, and the resulting variants were introduced into *E. coli* carrying *P_ttgABC_:egfp* as sensing elements. The engineered TtgRs investigated are summarized in Table 1, and the effects that this genetic engineering has on TtgR were evaluated using a biosensor assay against diverse flavonoids and resveratrol.

### 3.4. Effects of Engineered TtgRs on the Biosensors

**Figure 4:**
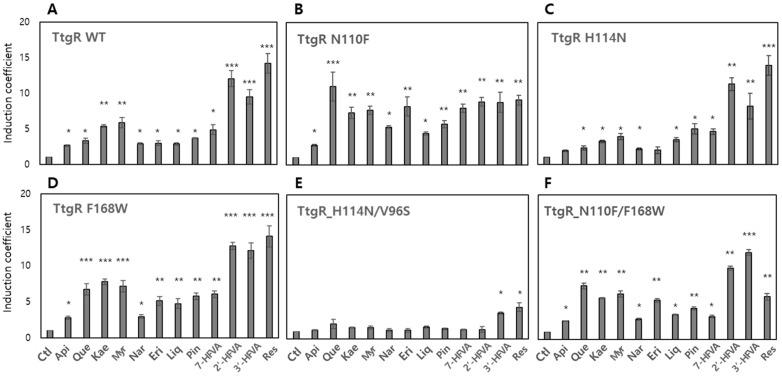
Responses of the *Escherichia coli* biosensors harboring wild-type (WT) and engineered TtgRs to 0.1 mM of the test compounds. Fluorescence responses are shown as induction coefficient values. (**A**–**F**) TtgR WT (**A**), N110F (**B**), H114N (**C**), F168W (**D**), H114N/V96S (**E**), and N110F/F168W (**F**). The data were replicated more than three times and asterisks indicate significant differences in data compared to the control (* *p* ≤ 0.05, ** *p* ≤ 0.01, and *** *p* ≤ 0.001 using Dunnett’s test).

Similarly to the TtgR WT, engineered TtgR variants were introduced into *E. coli* BL21 as sensing elements alongside the pTtg-eGFP reporter plasmid. Each biosensor was subjected to fluorescence-based assays under the same conditions as those applied to the biosensor harboring the TtgR WT. Among the tested TtgR variants, some exhibited a complete loss of responsiveness and selectivity toward the tested compounds, whereas others displayed altered sensing profiles compared with that of the TtgR WT (Figure 4 and Figure S2). These observations suggest that the conformational changes introduced through site-directed mutagenesis may have affected either the ligand-binding affinity or DNA-binding activity of TtgR as a transcriptional repressor. To validate these effects, biosensors expressing the engineered TtgRs were systematically evaluated for their responses to various flavonoids and resveratrol.

#### 3.4.1. Biosensor-TtgRs with Mutated Asn110

Among the residues targeted for mutagenesis, Asn110 near the B ring of flavonoids is located at the entrance of the binding pocket. To modulate the performance of the biosensors, Asn110 was mutated to tyrosine (Tyr; Y), Phe (F), and alanine (Ala; A), and these mutants were named TtgR N110Y, TtgR N110F, and TtgR N110A, respectively. They were introduced into *E. coli* with pTtg-eGFP, whereafter their target selectivity and specificity for 0.1 mM chemicals were investigated using a biosensor assay. Results showed that the TtgR N110Y and N110N variants exhibited similar patterns to that of the TtgR WT, with smaller induction coefficient values (Figure S2A,B). In the TtgR WT, the side chain of Asn110 is involved in interactions with the 3′- or 4′-OH of flavonoids via hydrogen bonding. In this regard, the biosensor-TtgR N110Y was expected to show similar responses to those of the TtgR WT because the Tyr side chain might preserve hydrogen bonding. However, the results indicated that hydrogen bonding is likely not the only factor affecting ligand interactions. Another factor contributing to the responses of biosensors could be determined from the background eGFP signal analysis, which is the arbitrary unit of eGFP obtained from the biosensors without chemical exposure (Figure S3). The biosensors with TtgR N110L and N110Y variants showed signals that were approximately eight and four times stronger than those of the TtgR WT. Genetic engineering likely affected ligand interactions, as well as interactions with DNA. Thus, functions as repressors should be considered to enhance the performance of TF-based biosensors.

In contrast, the biosensor-TtgR N110F showed significantly varied responses to the chemicals (Figure 4B). The responses to quercetin, kaempferol, and eriodictyol increased by more than two times, and the response to resveratrol decreased. The bulky and hydrophobic side chain of tryptophan (Trp) might have affected the position of ligands in the binding site, or π–π interactions with flavonoids might have contributed to the strong responses observed. Although further investigation is needed to elucidate these mechanisms, genetic engineering of TtgR clearly affected the selectivity and specificity of the biosensors. Moreover, the biosensor-TtgR N110F showed a 1.7 times increased background signal, thereby causing minor interference with TtgR as a repressor (Figure S3).

#### 3.4.2. Biosensor-TtgRs with Mutated His114

To investigate the role of His114 in ligand recognition, the TtgR mutants, H114A and H114N, were subjected to biosensor assays to assess their effects on selectivity and specificity. His114 contributes to ligand binding through hydrogen bonding, particularly in the lower region of the ligand-binding pocket (Figure S1). The biosensor harboring TtgR H114A exhibited a response profile similar to that of the TtgR WT but with a generally reduced signal intensity (Figure S2C), whereas TtgR H114N showed comparable response patterns and magnitudes with those of the WT (Figure 4C). These results suggest that the substitution of His114 with Ala abolished hydrogen bonding interactions with hydroxyl groups on the flavonoid B ring, whereas Asp substitution retained the capacity for hydrogen bonding. With respect to repressor function, TtgR H114A did not significantly affect DNA-binding activity, whereas TtgR H114N appeared to weaken DNA interactions, as evidenced by the elevated background fluorescence signals measured (Figure S3).

#### 3.4.3. Biosensor-TtgRs with Mutated Val96 and Ile141

In contrast to the previously described TtgR mutants, the genetic engineering of Val96 and Ile141—located in the upper region of the ligand-binding pocket—targeted residues that contribute to the formation of a hydrophobic cavity accommodating the A and C rings of flavonoids. Biosensors expressing the TtgR variants, V96S, V96F, and I141S, were evaluated for their responses to the test compounds. Mutations at these positions significantly attenuated biosensor responsiveness, with Val96 mutants particularly leading to a complete loss of both specificity and selectivity for flavonoids and resveratrol (Figure S3E,F). These findings highlight the importance of hydrophobicity and steric complementarity in the upper binding region for effective ligand recognition. Although the I141S mutant retained some responsiveness, its signal intensity and selectivity were markedly reduced (Figure S2G). This may be attributed to the introduction of a hydroxyl group from serine, which could disrupt the hydrophobic microenvironment necessary for stable ligand binding. In the case of V96F, despite preserving the hydrophobic character, the increased side chain bulk may have altered the shape of the binding pocket, thereby compromising ligand fit. Taken together, these results emphasize that both the hydrophobic nature and spatial architecture of the upper binding site in TtgR are essential determinants of ligand selectivity and specificity, particularly for the recognition of flavonoid structures.

#### 3.4.4. Biosensor-TtgRs with Phe168 and Double Mutations

The biosensor harboring the TtgR F168W variant maintained overall specificity and selectivity for the tested compounds, which were comparable with those of the TtgR WT biosensor (Figure 4D). Phe168 is located in the upper region of the ligand-binding pocket and contributes to the formation of a hydrophobic patch. Given that Trp is similarly hydrophobic but bulkier than Phe is, this substitution did not drastically alter the performance of the biosensor. However, a slight increase in responsiveness to 2′- and 3′-HFVA were observed, as well as to resveratrol (Figure 4D). This suggests that the narrow hydrophobic pocket formed by the bulkier Trp side chain may provide a better fit for these compounds. Importantly, the F168W mutation did not compromise the repressor function of TtgR, as evidenced by the background fluorescence levels measured that were comparable with those of the WT (Figure S3).

Among the double mutants, the biosensor expressing TtgR H114N/V96S completely lost responsiveness to the tested compounds (Figure 4E), indicating the likely disruption in both ligand interaction and structural integrity of the binding pocket. In contrast, the biosensor with the TtgR N110F/F168W variant displayed altered response patterns with an approximately twofold increase in background fluorescence (Figure 4F and Figure S3). Notably, the response to resveratrol was reduced compared with that of the WT, which may be attributed to the loss of hydrogen bonding interactions due to the N110F substitution.

### 3.5. Computational Analysis of TtgR–Ligand Interactions

**Figure 5:**
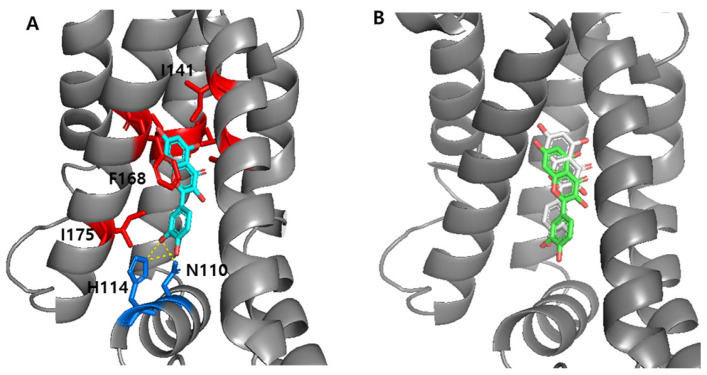
Docking model of the TtgR monomer–quercetin complex. (**A**) Quercetin (cyan) was stabilized by hydrophobic residues (L92, C137, I141, M167, F168, I175; red) and hydrogen bonds with N110 and H114 (blue). (**B**) Superimposed structures of the quercetin docked model (green) and crystal structure (gray), showing high structural alignment.

The crystal structure of the TtgR–quercetin complex (PDB ID: 2UXH) was used as a template for structural analysis. To prepare the protein for docking experiments with various ligands, quercetin was removed from the complex to generate the apo form of TtgR. To validate the docking protocol, quercetin was redocked into the binding site of the apo-TtgR WT (Figure 5A). The redocked quercetin adopted a binding pose highly similar to that observed in the original crystal structure, supporting the reliability of the docking methodology (Figure 5B). Subsequently, the interactions between ligands and engineered TtgR variants were analyzed using computational tools to investigate the altered ligand responses of the modified proteins.

#### 3.5.1. Interaction Between the TtgR Wild-Type and Ligands

The biosensor assays demonstrated that the TtgR WT had a broad specificity for the tested compounds, displaying particularly strong responses to resveratrol, 2′- and 3′-HFVA, quercetin, and kaempferol, among the flavonoids (Figure 3B). The structural analysis of the TtgR-binding site revealed that the A and C rings of quercetin are positioned within a hydrophobic patch, whereas the hydroxyl groups on the B ring form hydrogen bonds with key residues, notably His114 and Asn110, located in the lower region of the binding pocket.

**Figure 6:**
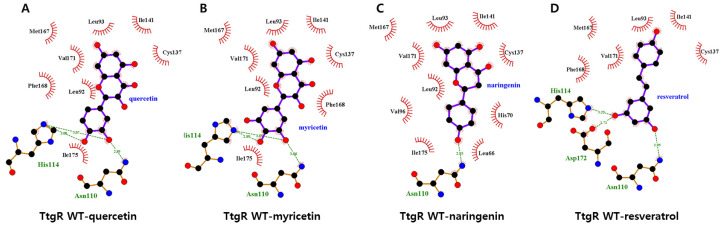
LigPlot analysis of the TtgR wild-type (WT) interactions with representative compounds. (**A**–**D**) Quercetin (**A**), myricetin (**B**), naringenin (**C**), and resveratrol (**D**). Hydrophobic interactions are indicated by red semicircles; hydrogen bonds are shown as green lines.

To investigate the molecular basis for the broad specificity observed, docking simulations were performed with apo-TtgR and selected ligands, including quercetin, myricetin, naringenin, and resveratrol (Figure 6). All flavonoids, except resveratrol, occupied similar positions in the binding pocket and consistently formed hydrogen bonds with His114 and Asn110. Naringenin, which exhibited a relatively weak biosensor response, formed a single hydrogen bond with Asn110. This reduced hydrogen bonding may explain the lower binding affinity and correspondingly weaker activation of the biosensor. Binding affinities derived from docking calculations supported these observations: myricetin exhibited the strongest binding with a predicted affinity of −8.1 kcal/mol, which was slightly higher than that of quercetin (−7.9 kcal/mol) and naringenin (−7.7 kcal/mol). These results suggest that both the position and number of hydroxyl groups on the B ring, particularly those capable of engaging in hydrogen bonding with His114 and Asn110, are critical for the effective release of TtgR from the promoter region.

In contrast, resveratrol, which elicited the strongest biosensor signal, occupied a distinct binding mode (Figure 6D); it was positioned deeper in the binding pocket and formed multiple, shorter hydrogen bonds with His114, Asn110, and Asp172. Additionally, the nonplanar structure of resveratrol may allow for a better fit into the hydrophobic patch than the planar flavonoid structures can, contributing to its enhanced binding and biosensor activation. Similar trends were observed for 2′- and 3′-HFVA, which exhibited strong biosensor responses (Figure 3B). Despite docking in orientations comparable with those of flavonoids, these flavanones positioned their A and C rings within the hydrophobic patch and formed hydrogen bonds through the hydroxyl groups of the B ring with residues in the lower binding region. However, other structurally related flavones showed no significant biosensor responses, implying that subtle structural differences—particularly molecular planarity—may influence the fit and interaction dynamics within the binding site.

Collectively, these results suggest that three main factors govern the interaction between TtgR and flavonoid ligands as follows: (1) hydrophobic interactions with the upper binding pocket, (2) hydrogen bonding with residues in the lower region, particularly via hydroxyl groups on the B ring, and (3) the overall molecular conformation, with nonplanar structures demonstrating enhanced compatibility. Nonetheless, given the large and chemically promiscuous nature of the TtgR-binding site, which accommodates diverse ligands beyond flavonoids [13,24], these findings are not conclusive. To gain deeper insight into ligand recognition, further computational analyses were conducted on engineered TtgR variants that exhibited altered responses in the biosensor assays.

#### 3.5.2. Engineered TtgRs with Asn110 Mutations

Asn110 in the TtgR WT was identified as a critical residue for ligand recognition, primarily because of its ability to form hydrogen bonds with hydroxyl groups, particularly on the C ring of flavonoids. To investigate the impact that this residue has on ligand interactions and biosensor specificity, Asn110 was substituted with Phe, Tyr, and leucine (Leu) to generate the following three TtgR variants: TtgR-N110F, TtgR-N110Y, and TtgR-N110L, respectively. These engineered proteins were incorporated into biosensor systems, and their response profiles to selected flavonoids and stilbenoids were evaluated.

**Figure 7:**
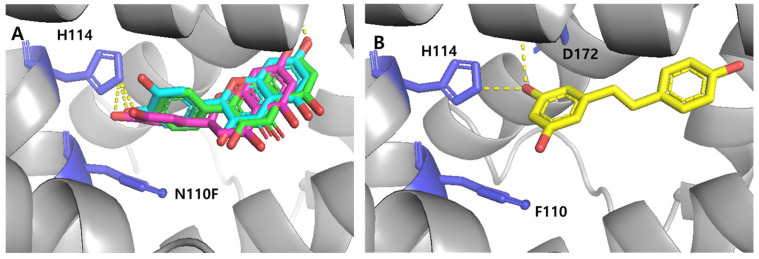
Docking analysis of the TtgR N110F variant with selected ligands. (**A**) Binding poses of quercetin (cyan), kaempferol (green), and eriodictyol (magenta), showing hydrogen bonds (yellow dotted lines) with H114 and π–π interactions with F110. (**B**) Binding pose of resveratrol (yellow), showing altered orientation when compared with that of the TtgR wild-type (WT).

Among the variants, biosensor TtgR-N110F displayed a markedly altered response profile. Specifically, it showed enhanced responses to several flavones, including quercetin, kaempferol, and eriodictyol, whereas the responses to flavanones and resveratrol were moderately reduced when compared with that of the WT. To elucidate the structural basis for these observations, molecular docking simulations were performed using the apo form of TtgR-N110F. Although substitution of Asn with Phe eliminated a key hydrogen bonding, it might introduce potential π–π stacking and enhanced hydrophobic interactions between Phe110 and aromatic moieties of the ligands. As shown in Figure 7A, quercetin, kaempferol, and eriodictyol—compounds that triggered stronger biosensor signals—were predicted to locate closely to Phe110. Although the possibility of π–π interactions was not experimentally validated in this study, it remains a plausible scenario supported by molecular simulations. Moreover, previous studies have reported that π–π stacking between polyphenols and aromatic residues contributes to the biological activity [36,37]. In the case of resveratrol, the replacement of Asn110 with Phe altered its docking pose and disrupted the hydrogen-bond network originally observed with His114, Asn110, and Asp172 (Figure 7B). This is likely to account for the reduced response of the TtgR-N110F biosensor. The N110Y and N110L variants did not confer any apparent benefits to ligand recognition. This may be due to the dual loss of hydrogen bonding capacity and lack of stabilizing π–π interactions, as neither Tyr nor Leu formed consistent noncovalent interactions with the tested ligands based on computational analysis.

Together, these results support the hypothesis that both hydrogen bonding (as provided by Asn110 in the WT) and π–π interactions (introduced by Phe110) play key roles in modulating ligand binding and biosensor responsiveness. The ability of Phe110 to enhance interactions with planar flavonoid structures may explain the increased selectivity toward specific flavones observed, whereas the diminished binding of nonplanar compounds, such as flavanones and resveratrol, underscores the structural constraints imposed by the mutated binding pocket.

#### 3.5.3. Engineered TtgRs with His114 and Phe168 Mutations

In addition to the N110 variants, biosensor assays using TtgR variants harboring substitutions at His114 and Phe168 revealed altered responses to selected flavonoids. To gain insight into the molecular basis for these modulated responses, ligand–protein interactions were analyzed using computational docking, focusing on hydrogen bonding and hydrophobic interactions within the binding pocket. Quercetin and resveratrol, as representative ligands, were docked into three-dimensional structures of the TtgR-H114 and TtgR-F168 variants, as modeled using PyMOL (Figure S4).

Substitutions at His114 and Phe168 influenced ligand binding through distinct yet complementary mechanisms. The H114N variant retained its hydrogen bonding capacity via its polar side chain, resulting in ligand-binding patterns and biosensor responses similar to those of the WT. In contrast, the H114A mutation disrupted key hydrogen bonds in the lower binding region, weakening ligand interactions and reducing the sensor output. The F168W substitution, however, introduced a bulkier aromatic residue that reshaped the hydrophobic pocket, providing potential π–π stacking and promoting more favorable orientations for aromatic ligands. These structural changes contributed to increased biosensor responses to flavonoids, such as quercetin, kaempferol, and galangin, and facilitated better accommodation of resveratrol due to its nonplanar structure.

Together, these findings highlight the distinct roles that hydrogen bonding and hydrophobic interactions play in TtgR-mediated ligand recognition. Although residue 114 governs interaction strength through polar contacts, modifications at residue 168 tune the spatial fit and stabilize forces within the pocket. Consistency between the docking models and biosensor data supports a structure–function relationship that explains the altered specificity and sensitivity observed in the engineered TtgR variants.

### 3.6. Quantification of Quercetin and Resveratrol Using the TtgR-Based Biosensors

The *ttg* operon-based whole-cell biosensors were evaluated for their ability to quantify quercetin and resveratrol. The engineered variant, TtgR N110F, which exhibited enhanced responsiveness to flavones, was used for quercetin detection, whereas the TtgR WT was used for resveratrol detection. Calibration curves were generated by measuring the fluorescence responses across a concentration range of 0–5 mM, with linear regression analysis applied within the linear response range (0–0.1 mM) to derive standard equations for quantification (Figure S5A,B). Experimental samples containing 10 and 15 mM of each compound were diluted to fit within this linear range, and their concentrations were estimated using the respective calibration equations. To validate the biosensor-based quantification, the same samples were analyzed in parallel using HPLC. The HPLC calibration curves were constructed using identical standard solutions (Figure S5C,D), and the concentrations were determined from the peak area integration. As summarized in Table 3, concentrations derived from the biosensor data showed a strong agreement with those obtained from HPLC, with quantification accuracies ranging from 96–98%.

## 4. Discussion

The transcription factor TtgR from *Pseudomonas putida* has been widely studied for its role in multidrug resistance regulation and its ability to respond to a broad range of structurally diverse compounds. In this study, we leveraged TtgR and its engineered variants to construct flavonoid-responsive whole-cell biosensors. The biosensors used eGFP as a reporter gene under the control of the TtgR-repressible *ttgABC* promoter (P*_ttgABC_*), enabling quantification of ligand-responsive gene expression.

The biosensor containing TtgR WT showed the strongest response toward resveratrol and to certain flavanones with hydroxyl groups in the B ring, while responses to flavones were weaker (Figure 4A). Because the specificity and sensitivity of TF-based biosensors are determined by the transcription factor itself, we applied structure-guided mutagenesis to key residues in the binding pocket, including Asn110, His114, and Phe168 (Figure 5). These residues were selected for their involvement in ligand recognition via hydrogen bonding and hydrophobic complementarity. Thus, we examined a series of TtgR variants with amino acid substitutions at these key residues and surrounding sites. As described above, not all mutations improved performance in a predictable way (Figure 4 and Figure S2). Among the tested variants, N110F substitution greatly enhanced the response to quercetin, increasing the dynamic range and lowering the detection limit, probably due to additional π–π stacking with the ligand [36,37]. N110Y, despite having an aromatic side chain, showed a different profile, indicating that steric and electronic effects also contribute to ligand recognition. Mutations close to the DNA-binding domain or dimerization interface often reduce DNA-binding affinity or protein stability, leading to higher background fluorescence and lower responsiveness.

**Figure 8:**
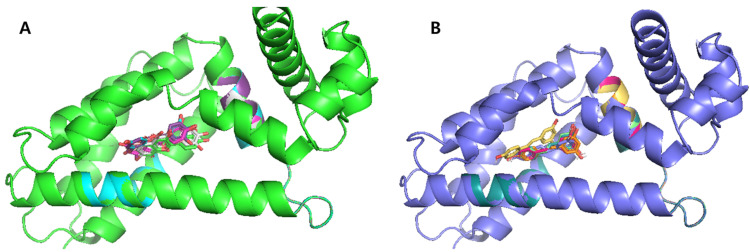
Superimposed structures of TtgR variants complexed with quercetin (**A**) and resveratrol (**B**). The TtgR wild-type (WT), H114A, H114N, N110F, N110Y, F168W, and N110F/F168W variants are compared. While the overall protein conformations remain conserved, the ligand binding poses varied depending on the mutations.

To better understand the differences among variants, we performed molecular docking and structural modeling. The superimposition of WT and mutant structures bound to quercetin or resveratrol showed little change in the protein backbone but clear differences in ligand orientation (Figure 8). In WT TtgR, both ligands were stabilized by hydrogen bonds to Asn110 and hydrophobic contacts with Phe168. In N110F, the aromatic phenyl group promoted stronger π–π stacking, pulling the ligand deeper into the hydrophobic pocket and altering hydrogen bond geometry. Substitutions at His114 often disrupted hydrogen bonding with resveratrol, weakening its stabilization. Changes in the upper hydrophobic patch usually did not improve selectivity and sometimes weakened interactions by disrupting key hydrophobic contacts. In contrast, changes in the lower pocket (N110F, H114N, and F168W) more effectively altered ligand orientation and specificity.

After experimentally assessing the responses of the biosensors to various flavonoids and resveratrol, we performed computational docking analyses to better understand the relationship between structural modifications in TtgR and the performance of biosensors. These analyses focused on the hydrogen bonding and hydrophobic interactions between TtgR variants and ligands and ligand orientation within the binding pocket to account for the observed behaviors of biosensors. Although these combined approaches provided valuable insights, accurately predicting biosensor performance from structural data alone remains challenging due to the complex interplay of multiple factors of transcription factor function, which involves not only ligand affinity but also protein stability, DNA-binding dynamics, and allosteric effects. Nevertheless, our experimental results allowed us to identify key trends linking specific substitutions to changes in selectivity and sensitivity. These findings, together with the proposed mechanistic explanations, are summarized in Table 4, which provides a concise overview of how each TtgR variant influences overall biosensor performance. The table lists the induction coefficient values for resveratrol, quercetin, and the additional ligand showing the strongest response for each variant. While the proposed mechanistic explanations have not yet been fully validated, they are derived from both experimental results and computational analyses.

To validate the applicability of the biosensors, biosensor-TtgR WT and biosensor-TtgR N110F were applied to quantify resveratrol and quercetin, respectively. When compared with HPLC analysis, both biosensors achieved a detection accuracy of over 90%. Based on calibration curves, the detection limit for the TtgR WT sensor was calculated as 0.0037 mM for resveratrol, and that for the N110F variant was 0.0063 mM for quercetin. These values are comparable to those reported for flavonoid biosensors based on FdeR or QdoR and fall within the concentration ranges typically observed in microbial production strains [38]. Such sensitivity is suitable for the in vivo monitoring of flavonoid production in metabolic engineering.

In other TF-based biosensors, such as those employing ArsR, ZntR, or MarR, IPTG induction from T7 promoters is often used to increase TF expression and reduce background leakage [39,40,41]. However, in the present study, IPTG induction did not improve sensor performance. On the contrary, IPTG supplementation slightly inhibited cell growth and reduced sensing efficiency. We found that leaky expression from the T7 promoter without IPTG was sufficient to maintain strong repression in the WT sensor, as evident from the low basal eGFP signals (Figure 3A and Figure S3). This suggests that, at least for TtgR, the native leaky expression level provides an optimal balance between sufficient repressor abundance and minimal metabolic burden. The finding also implies that excessive TF levels may not always correlate with better repression or sensitivity, as high expression could perturb folding, stability, or DNA-binding kinetics.

Although the need for flavonoid-targeted biosensors may seem limited, their potential applications are expanding. Similar systems have been used for the directed evolution, high-throughput screening, and optimization of biosynthetic pathways. In such contexts, the ability to monitor flavonoid production in real time offers a valuable alternative to labor-intensive analytical methods. Furthermore, the broad recognition range of TtgR provides opportunities to detect different classes of polyphenols, making it a flexible platform for pathway screening in synthetic biology.

In conclusion, TtgR’s broad ligand recognition makes it a versatile scaffold but poses challenges for engineering high selectivity. Small, targeted substitutions can adjust ligand orientation and interaction patterns without major structural changes. A deeper understanding of TtgR–ligand interactions will help in designing customized biosensors with improved selectivity and sensitivity. With further optimization in TF engineering, expression control, and reporter configuration, TtgR-based systems could serve as low-cost, adaptable tools for metabolic engineering, synthetic biology, and the detection of polyphenolic compounds [15,18,42].

## Figures and Tables

**Table 1:** Genetic characteristics of the plasmids and *Escherichia coli* cell-based biosensors used in this study.

	Name	Description	Reference
Plasmids	pET-21a(+)	pBR322 ori, Amp ^r^	Novagen
	pCDF-Duet	CloDE13 ori, Str ^r^	Novagen
	pTtg-eGFP	pET-21a(+) carrying P*_ttgABC_:egfp*	This study
	pCDF-TtgR WT	pCDF-Duet carrying *ttgR* WT	This study
	pCDF-TtgR mut	pCDF-Duet carrying *ttgR* mutants: N110F, N110L, N110Y, H114N, H114A, V96S, I141S, I141L, F168W, N110Y/F168W, V96S/H114N	This study
			
			
*E. coli* strains	*E. coli* BL21 (DE3)	F^−^ *ompT hsdS_B_*(r_B_^−^m_B_^−^)*gal dcm lon* (DE3)	Stratagene
	biosensor-TtgR WT	*E. coli* BL21 carrying pCDF-TtgR WT and pTtg-eGFP	This study
	biosensor-TtgR muts	*E. coli* BL21 carrying pCDF-TtgR muts and pTtg-eGFP	This study

eGFP, enhanced green fluorescent protein; mut, mutant; WT, wild-type.

**Table 2:** The compounds tested for flavone, flavanone and resveratrol.

	Abbreviation	Full Name	3	5	6	7	8	2′	3′	4′	5′	6′
Flavone	Lut	Luteolin	H	OH	H	OH	H	H	OH	OH	H	H
	Api	Apigenin	H	OH	H	OH	H	H	H	OH	H	H
	Que	Quercetin	OH	OH	H	OH	H	H	OH	OH	H	H
	Kae	Kaempferol	OH	OH	H	OH	H	H	H	OH	H	H
	Myr	Myricetin	OH	OH	H	OH	H	H	OH	OH	OH	H
	Gal	Galangin	OH	OH	H	OH	H	H	H	H	H	H
	4′-HFV	4′-hydroxyflavone	H	H	H	H	H	H	H	OH	H	H
	3′-HFV	3′-hydroxyflavone	H	H	H	H	H	H	OH	H	H	H
	2′-HFV	2′-hydroxyflavone	H	H	H	H	H	OH	H	H	H	H
	5-HFV	5-hydroxyflavone	H	OH	H	H	H	H	H	H	H	H
	7-HFV	7-hydroxyflavone	H	H	H	OH	H	H	H	H	H	H
	7,3′-DHFV	7,3′-dihydroxyflavone	H	H	H	OH	H	H	OH	H	H	H
Flavanone	Nar	Naringenin	H	OH	H	OH	H	H	H	OH	H	H
	Eri	Eriodictyol	H	OH	H	OH	H	H	OH	OH	H	H
	Liq	Liquiritigenin	H	H	H	OH	H	H	H	OH	H	H
	Pin	Pinocembrin	H	OH	H	OH	H	H	H	H	H	H
	3′-HFVA	3′-hydroxyflavanone	H	H	H	H	H	H	OH	H	H	H
	2′-HFVA	2′-hydroxyflavanone	H	H	H	H	H	OH	H	H	H	H
	7-HFVA	7-hydroxyflavanone	H	H	H	OH	H	H	H	H	H	H
Stilbene	Res	Resveratrol										

**Table 3:** Quantification of resveratrol and quercetin using the *Escherichia coli* cell-based biosensors and HPLC.

Methods	Samples	Tested Conc. (mM)	Measured Conc. (mM)	Accuracy (%)
HPLC	Resveratrol	10	10.12 ± 0.59	98.8
		15	15.34 ± 0.48	97.8
	Quercetin	10	9.8 ± 0.20	98.0
		15	15.62 ± 0.93	96.0
Biosensor-TtgR WT	Resveratrol	10	9.55 ± 0.64	95.5
		15	14.24 ± 0.86	94.9
Biosensor-TtgR N110F	Quercetin	10	9.36 ± 0.25	93.6
		15	14.42 ± 0.52	96.1

Conc., concentration; HPLC, high-performance liquid chromatography; WT, wild-type.

**Table 4:** Summary of performance profiles and proposed mechanistic explanations for TtgR-based biosensors.

TtgRs	I.C of Main Ligands	Detection Limit (mM)	Basal eGFP Signal	Proposed Mechanistic Reasons
WT	Resveratrol (12.4)	0.0037	Low	Native binding pocket optimized for resveratrol; strong DNA-binding repression
	2′-HFVA (10.9)			
	Quercetin (2.9)			
N110F	Quercetin (11.0)	0.0063	Low–Moderate	Aromatic Phe substitution enhances π–π stacking with quercetin; increases conformational flexibility
	Resveratrol (9.1)			
	2′-HFVA (8.8)			
N110Y	Resveratrol (9.3)	-	Moderate	Bulkier Tyr may improve aromatic interactions but also cause steric hindrance
	3′-HFVA (8.2)			
	Quercetin (2.1)			
H114N/V96S	Resveratrol (4.4)	-	Moderate	Loss of aromaticity reduces π–π interactions; possible altered pocket shape
	3′-HFVA (3.6)			
	Quercetin (2.1)			
N110L	2′-HFVA (8.5)	-	Low	Hydrophobic Leu substitution maintains repression; minimal effect on ligand accommodation
	Resveratrol (7.2)			
	Quercetin (3.1)			
N110F/F168W	2′-HFVA (12.0)	-	Low	Similar polarity to WT; minor structural change
	Quercetin (7.4)			
	Resveratrol (5.9)			
H114A	Resveratrol (7.0)	-	Moderate–High	Removal of imidazole side chain disrupts hydrogen bonding
	2′-HFVA (4.0)			
	Quercetin (3.1)			
H114N	Resveratrol (11.8)	-	Moderate	Maintains hydrogen bonding potential but alters geometry
	2′-HFVA (8.3)			
	Quercetin (4.6)			
V96F	Resveratrol (2.2)	-	Moderate	Bulky aromatic substitution changes hydrophobic packing near ligand site
	2′-HFVA (2.0)			
	Quercetin (1.6)			
V96S	Resveratrol (5.8)	-	Moderate–High	Polar Ser alters hydrophobic microenvironment; possible reduced ligand affinity
	2′-HFVA (3.7)			
	Quercetin (2.1)			
I141S	Resveratrol (6.8)	-	High	Polar substitution at hydrophobic site destabilizes closed conformation
	Myricetin (5.5)			
	Quercetin (3.2)			
F168W	Resveratrol (14.1)	-	Low–Moderate	Larger aromatic Trp modifies π–π stacking geometry and steric profile
	2′-HFVA (12.8)			
	Quercetin (6.7)			

Detection limit: Values for TtgR WT (0.0037 mM, resveratrol) and TtgR N110F (0.0063 mM, quercetin) were determined from calibration curves as LOD = 3 × (standard deviation of blank)/slope, and they correspond to the lowest concentration giving a signal significantly above the control (*p* < 0.05). Basal eGFP signal classification: “Narrow”, “Moderate”, or “Broad”, defined based on fold change in eGFP signal between control and ligand at 0.1 mM.

## Data Availability

The original contributions presented in this study are included in the article/Supplementary Materials. Further inquiries can be directed to the corresponding author(s).

## Supplementary Materials

The following supporting information can be downloaded at: https://www.mdpi.com/article/10.3390/bios15080554/s1, Table S1: List of primers used for cloning PttgABC and ttgR variants; Figure S1: Three-dimensional structure of the TtgR WT dimer in complex with quercetin, highlighting the residues involved in ligand interactions within the binding pocket; Figure S2: Responses of the Escherichia coli biosensors harboring engineered TtgRs to 0.1 mM of the test compounds. Fluorescence responses are shown as induction coefficient values. TtgR N110Y (A), N110L (B), H114A (C), V96S (D), V96F (E), I141S; Figure S3: Background fluorescence signals of biosensors harboring wild-type and engineered TtgRs; Figure S4: Docking poses of quercetin (cyan) and resveratrol (yellow) in TtgR variants: H114A (A), H114N (B), and F168W (C); Figure S5: Standard curves for the quantification of quercetin and resveratrol using HPLC and TtgR-based biosensors.

## References

[1] Sun J., Deng Z., Yan A. (2014). Bacterial multidrug efflux pumps: Mechanisms, physiology and pharmacological exploitations. Biochem. Biophys. Res. Commun..

[2] Daniels C., Daddaoua A., Lu D., Zhang X., Ramos J.-L. (2010). Domain cross-talk during effector binding to the multidrug binding TTGR regulator. J. Biol. Chem..

[3] Blanco P., Hernando-Amado S., Reales-Calderon J.A., Corona F., Lira F., Alcalde-Rico M., Bernardini A., Sanchez M.B., Martinez J.L. (2016). Bacterial multidrug efflux pumps: Much more than antibiotic resistance determinants. Microorganisms.

[4] Grkovic S., Brown M.H., Roberts N.J., Paulsen I.T., Skurray R.A. (1998). QacR is a repressor protein that regulates expression of theStaphylococcus aureus multidrug efflux pump QacA. J. Biol. Chem..

[5] Terán W., Felipe A., Segura A., Rojas A., Ramos J.-L., Gallegos M.A.-T. (2003). Antibiotic-dependent induction of Pseudomonas putida DOT-T1E TtgABC efflux pump is mediated by the drug binding repressor TtgR. Antimicrob. Agents Chemother..

[6] Klyachko K.A., Schuldiner S., Neyfakh A.A. (1997). Mutations affecting substrate specificity of the Bacillus subtilis multidrug transporter Bmr. J. Bacteriol..

[7] Alguel Y., Meng C., Terán W., Krell T., Ramos J.L., Gallegos M.-T., Zhang X. (2007). Crystal structures of multidrug binding protein TtgR in complex with antibiotics and plant antimicrobials. J. Mol. Biol..

[8] Terán W., Krell T., Ramos J.L., Gallegos M.-T. (2006). Effector-repressor interactions, binding of a single effector molecule to the operator-bound TtgR homodimer mediates derepression. J. Biol. Chem..

[9] Wu Y., Zhang S., York D.M., Wang L. (2024). Adsorption of Flavonoids in a Transcriptional Regulator TtgR: Relative Binding Free Energies and Intermolecular Interactions. J. Phys. Chem. B.

[10] Dias M.C., Pinto D.C., Silva A.M. (2021). Plant flavonoids: Chemical characteristics and biological activity. Molecules.

[11] Pourcel L., Routaboul J.-M., Cheynier V., Lepiniec L., Debeaujon I. (2007). Flavonoid oxidation in plants: From biochemical properties to physiological functions. Trends Plant Sci..

[12] Kumar S., Pandey A.K. (2013). Chemistry and biological activities of flavonoids: An overview. Sci. World J..

[13] Fernandez-Escamilla A.M., Fernandez-Ballester G., Morel B., Casares-Atienza S., Ramos J.L. (2015). Molecular binding mechanism of TtgR repressor to antibiotics and antimicrobials. PLoS ONE.

[14] Paczkowski J.E., Mukherjee S., McCready A.R., Cong J.-P., Aquino C.J., Kim H., Henke B.R., Smith C.D., Bassler B.L. (2017). Flavonoids suppress Pseudomonas aeruginosa virulence through allosteric inhibition of quorum-sensing receptors. J. Biol. Chem..

[15] Espinosa-Urgel M., Serrano L., Ramos J.L., Fernández-Escamilla A.M. (2015). Engineering biological approaches for detection of toxic compounds: A new microbial biosensor based on the Pseudomonas putida TtgR repressor. Mol. Biotechnol..

[16] Rogers J.K., Guzman C.D., Taylor N.D., Raman S., Anderson K., Church G.M. (2015). Synthetic biosensors for precise gene control and real-time monitoring of metabolites. Nucleic Acids Res..

[17] Chen W., Zhang X., Xiong D., Jin J.-M., Tang S.-Y. (2019). Engineering the effector specificity of regulatory proteins for the in vitro detection of biomarkers and pesticide residues. Appl. Microbiol. Biotechnol..

[18] Ding N., Zhou S., Deng Y. (2021). Transcription-factor-based biosensor engineering for applications in synthetic biology. ACS Synth. Biol..

[19] Pham C., Stogios P.J., Savchenko A., Mahadevan R. (2022). Advances in engineering and optimization of transcription factor-based biosensors for plug-and-play small molecule detection. Curr. Opin. Biotechnol..

[20] Jeon Y., Lee Y., Jang G., Kim B.-G., Yoon Y. (2022). Design of Pb (II)-Specific E. coli-based biosensors by engineering regulatory proteins and host cells. Front. Microbiol..

[21] Ghataora J.S., Gebhard S., Reeksting B.J. (2023). Chimeric MerR-family regulators and logic elements for the design of metal sensitive genetic circuits in Bacillus subtilis. ACS Synth. Biol..

[22] Li M., Chen Z., Huo Y.-X. (2024). Application evaluation and performance-directed improvement of the native and engineered biosensors. ACS Sens..

[23] Zhou G.J., Zhang F. (2023). Applications and tuning strategies for transcription factor-based metabolite biosensors. Biosensors.

[24] de Carvalho Matias E., Bezerra K., Costa A.L., Clemente Junior W., Oliveira J., Ribeiro Junior L., Galvão D., Fulco U. (2024). Quantum biochemical analysis of the TtgR regulator and effectors. Sci. Rep..

[25] Yoon Y., Kim S., Chae Y., Kim S.W., Kang Y., An G., Jeong S.-W., An Y.-J. (2016). Simultaneous detection of bioavailable arsenic and cadmium in contaminated soils using dual-sensing bioreporters. Appl. Microbiol. Biotechnol..

[26] DeLano W.L., Bromberg S. (2004). PyMOL User’s Guide.

[27] Morris G.M., Huey R., Lindstrom W., Sanner M.F., Belew R.K., Goodsell D.S., Olson A.J. (2009). AutoDock4 and AutoDockTools4: Automated docking with selective receptor flexibility. J. Comput. Chem..

[28] Trott O., Olson A.J. (2010). AutoDock Vina: Improving the speed and accuracy of docking with a new scoring function, efficient optimization, and multithreading. J. Comput. Chem..

[29] Pettersen E.F., Goddard T.D., Huang C.C., Couch G.S., Greenblatt D.M., Meng E.C., Ferrin T.E. (2004). UCSF Chimera—A visualization system for exploratory research and analysis. J. Comput. Chem..

[30] Wallace A.C., Laskowski R.A., Thornton J.M. (1995). LIGPLOT: A program to generate schematic diagrams of protein-ligand interactions. Protein Eng. Des. Sel..

[31] R Core Team (2013). R: A Language and Environment for Statistical Computing.

[32] Signorell A., Aho K., Alfons A., Anderegg N., Aragon T., Arppe A., Baddeley A., Barton K., Bolker B., Borchers H.W. (2019). DescTools: Tools for Descriptive Statistics.

[33] Nishikawa K.K., Hoppe N., Smith R., Bingman C., Raman S. (2021). Epistasis shapes the fitness landscape of an allosteric specificity switch. Nat. Commun..

[34] Song K., Ji H., Lee J., Yoon Y. (2025). Microbial Transcription Factor-Based Biosensors: Innovations from Design to Applications in Synthetic Biology. Biosensors.

[35] Ding N., Yuan Z., Zhang X., Chen J., Zhou S., Deng Y. (2020). Programmable cross-ribosome-binding sites to fine-tune the dynamic range of transcription factor-based biosensor. Nucleic Acids Res..

[36] Akher F.B., Ebrahimi A., Mostafavi N. (2017). Characterization of π-stacking interactions between aromatic amino acids and quercetagetin. J. Mol. Struct..

[37] Shahidi F., Dissanayaka C.S. (2023). Phenolic-protein interactions: Insight from in-silico analyses–a review. Food Prod. Process. Nutr..

[38] Siedler S., Stahlhut S.G., Malla S., Maury J., Neves A.R. (2014). Novel biosensors based on flavonoid-responsive transcriptional regulators introduced into Escherichia coli. Metab. Eng..

[39] Lee W., Kim H., Jang G., Kim B.-G., Yoon Y. (2020). Antimony sensing whole-cell bioreporters derived from ArsR genetic engineering. Appl. Microbiol. Biotechnol..

[40] Kim Y., Jeon Y., Jang G., Kim B.-G., Yoon Y. (2024). A novel Escherichia coli cell–based bioreporter for quantification of salicylic acid in cosmetics. Appl. Microbiol. Biotechnol..

[41] Kim H., Jang G., Kim B.-G., Yoon Y. (2020). Modulation of the metal (loid) specificity of whole-cell bioreporters by genetic engineering of ZntR metal-binding loops. J. Microbiol. Biotechnol..

[42] Kim N.M., Sinnott R.W., Sandoval N.R. (2020). Transcription factor-based biosensors and inducible systems in non-model bacteria: Current progress and future directions. Curr. Opin. Biotechnol..

