# Supplementary material for: Engineered TtgR-Based Whole-Cell Biosensors for Quantitative and Selective Monitoring of Bioactive Compounds
Source: Biosensors (Basel). 2025 Aug 21;15(8):554. doi: 10.3390/bios15080554 (PMC12384351; doi:10.3390/bios15080554)
Supplement: Supplementary file 1 [file biosensors-15-00554-s001.zip › biosensors-3774863-supplementary.pdf]

biosensors

*Supplementary Materials*

# Engineered TtgR-Based Whole-Cell Biosensors for Quantitative and Selective Monitoring of Bioactive Compounds

**Kyeongseok Song [1], Haekang Ji [1], Jiwon Lee [1], Geupil Jang [2] and Youngdae Yoon [1,*]**

[1] Department of Environmental Health Science, Konkuk University, Seoul 05029, Republic of Korea.
[2] School of Biological Sciences and Technology, Chonnam National University, Gwangju 61186, Republic of Korea.
* Correspondence: yyoon21@gmail.com; Tel.: +82-2-450-0443; Fax: +82-2-450-3726

**Table S1.** List of primers used for cloning of $P_{ttgABC}$ and *ttgR* variants. Restriction enzyme recognition sites and engineered mutation sites are underlined.

| Gene | Sequence (5′ to 3′) | restriction enz. |
|---|---|---|
| $P_{ttgABC}$ | GCGCAGATCTCCCAGCAGTATTTACAAACAA | *BglII* |
| | GCGCGCGCTCTAGAAAATAGCTTGCTAAGGAATATACTTAC | *XbaI* |
| TtgR | ATATACCATGGTCCGTCGAACCAAAGAAGA | *NcoI* |
| | CATTATGCGGCCGCTCATTTGCGCAGAGCC | *NotI* |
| TtgR N110F | GAACCCGTCGTATCTTCGAAATCCTGCATC | |
| | GATGCAGGATTTCGAAGATACGACGGGTTC | |
| TtgR N110L | CGAACCCGTCGTATCCTTGAAATCCTGCATC | |
| | GATGCAGGATTTCAAGGATACGACGGGTTCG | |
| TtgR N110Y | GAACCCGTCGTATCTATGAAATCCTGCATC | |
| | GATGCAGGATTTCATAGATACGACGGGTTC | |
| TtgR H114N | GTATCAATGAAATCCTGAATCACAAGTGCGAGTTC | |
| | GAACTCGCACTTGTGATTCAGGATTTCATTGATAC | |
| TtgR H114A | GTATCAATGAAATCCTGGCTCACAAGTGCGAGTTC | |
| | GAACTCGCACTTGTGAGCCAGGATTTCATTGATAC | |
| TtgR V96S | CAAGCTGCTGTTGCAATCGTTTAACGAGCTGGTG | |
| | CACCAGCTCGTTAAACGATTGCAACAGCAGCTTG | |
| TtgR I141S | CCACAAGGGCAAGTCCCTGGCGCTG | |
| | CAGCGCCAGGGACTTGCCCTTGTGG | |
| TtgR I141L | CTGGATTGCCACAAGGGCTTAACCCTGGCGCTGGCCAATG | |
| | CATTGGCCAGCGCCAGGGTTAAGCCCTTGTGGCAATCCAG | |
| TtgR F168W | CGGTGGCCATGTGGGCCTATGTCGATG | |
| | CATCGACATAGGCCCACATGGCCACCG | |

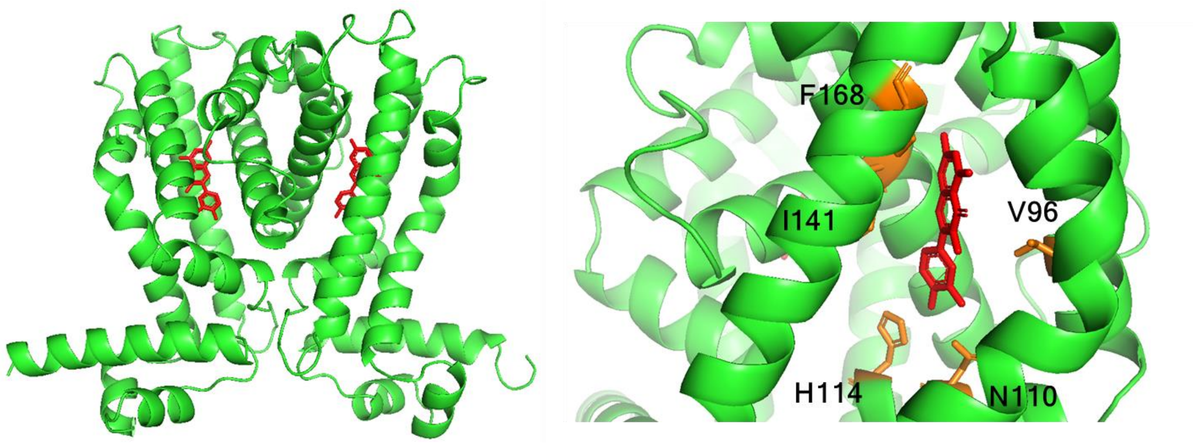


**Figure S1.** Three-dimensional structure of the TtgR WT dimer in complex with quercetin, highlighting the residues involved in ligand interactions within the binding pocket.

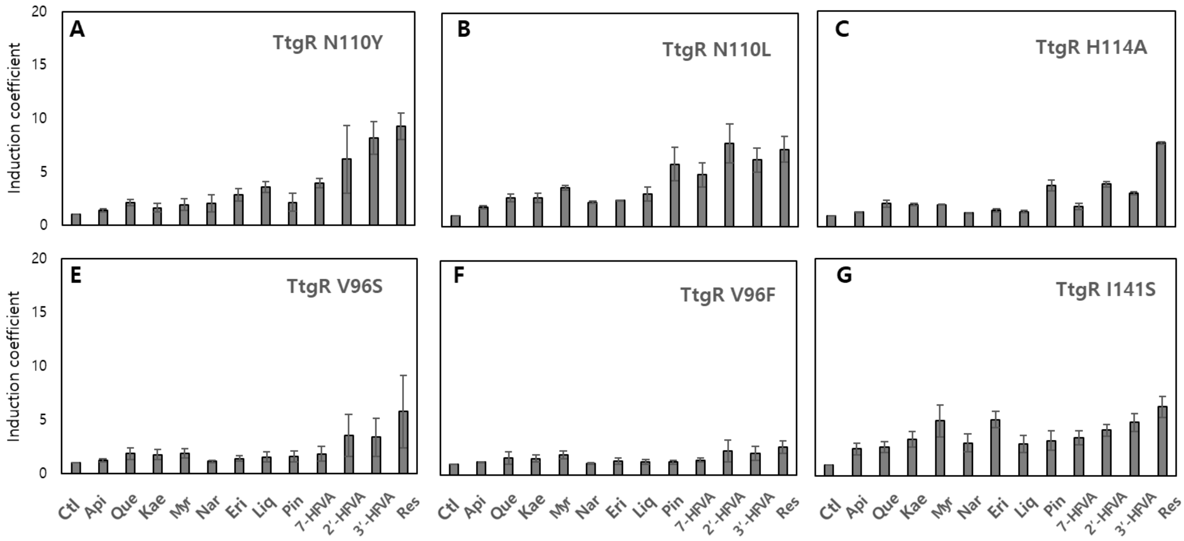


**Figure S2.** Responses of the *Escherichia coli* biosensors harboring engineered TtgRs to 0.1 mM of the test compounds. Fluorescence responses are shown as induction coefficient values. TtgR N110Y (A), N110L (B), H114A (C), V96S (D), V96F (E), I141S.

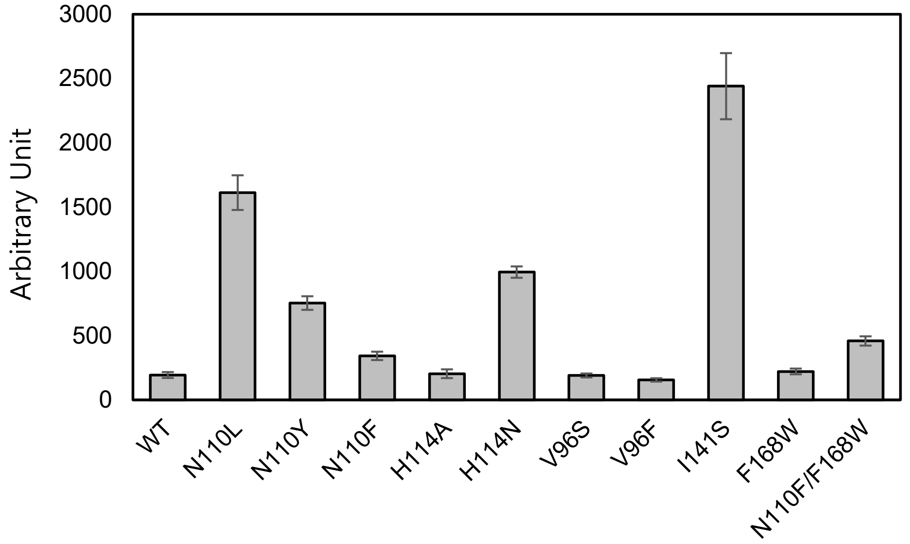


**Figure S3.** Background fluorescence signals of biosensors harboring wild-type and engineered TtgRs. eGFP intensities were measured in the absence of ligand exposure and are presented in arbitrary units using a fluorescence spectrophotometer.

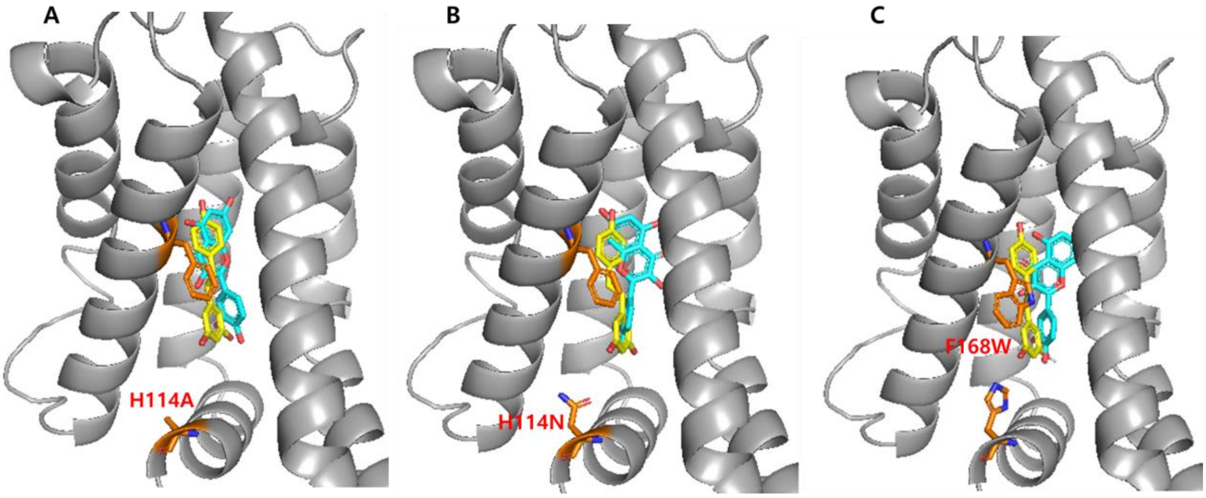


**Figure S4.** Docking poses of quercetin (cyan) and resveratrol (yellow) in TtgR variants: H114A (A), H114N (B), and F168W (C).

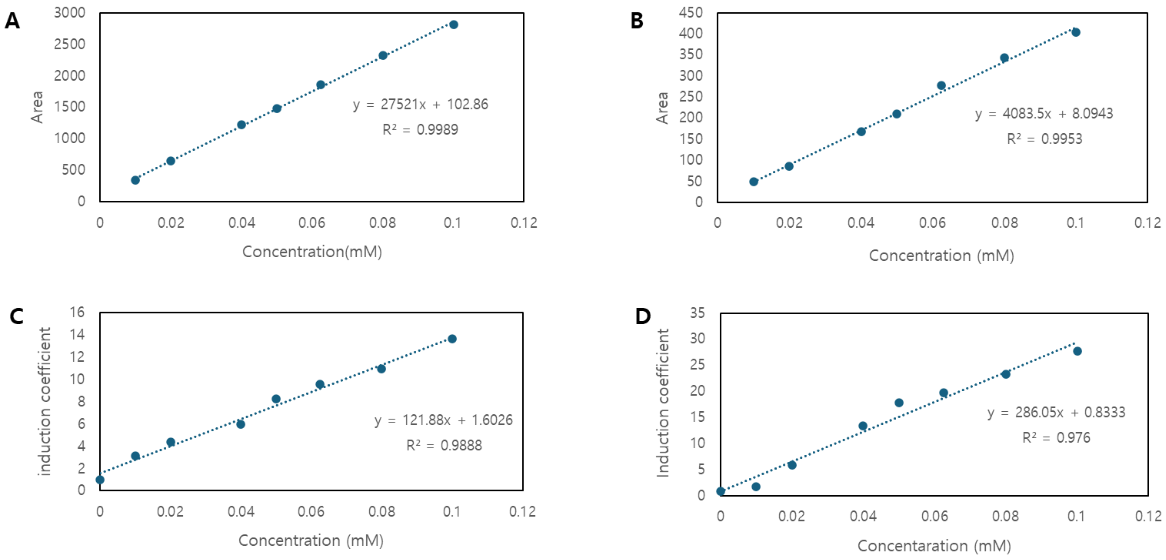


**Figure S5.** Standard curves for the quantification of quercetin and resveratrol using HPLC and TtgR-based biosensors. (A) Quercetin standard curve obtained by HPLC analysis, (B) Resveratrol standard curve obtained by HPLC analysis, (C) Quercetin standard curve generated using biosensor-TtgR N110F, (D) Resveratrol standard curve generated using biosensor-TtgR WT.
